# G-protein signaling modulator 1 is a modifier of sGC-dependent vascular response

**DOI:** 10.1186/2050-6511-14-S1-P11

**Published:** 2013-08-29

**Authors:** George Britton, Iraida Sharina, Emil Martin

**Affiliations:** 1 University of Texas Houston Medical School, Department of Internal Medicine, Division of Cardiology, Houston, Texas, USA

## Background

Earlier investigations demonstrated that the activity and function of soluble guanylyl cyclase (sGC) may be modulated by interacting proteins, such as HSP70, HSP90, CCTη or PDI. We have also previously demonstrated that G-protein signaling modulator proteins 1 and 2 (GPSM1, 2) directly interact with the catalytic region of sGC and attenuate the response of sGC to activators in cell lysates.

## Results

In this report, we provide evidence that vascular function of sGC is increased in GPSM1-deficient mice. We found that GPSM1-deficient mice have a lower resting blood pressure than their wild type counterparts. Intraperitoneal injection of sGC stimulator BAY 41-2272 elicited a transient decrease in blood pressure in both strains. However, GPSM1^-/-^ mice were more sensitive to lower doses of BAY 41-2272, while the decrease in blood pressure was more profound and more sustained than in wild type mice. In ex vivo setting, preconstricted aortic rings from GPSM1^-/-^ mice were more sensitive to acetylcholine and BAY 41-2272. Western blotting showed similar level of sGC expression in aortas of both strains. H&E staining of aorta sections showed no obvious morphologic differences between these strains of mice. However, aortas from GPSM1^-/-^ mice sowed a higher level of cGMP accumulated in response to NO donor DEA-NO than from wild type animals. Unexpectedly, cGMP-degrading activity was also higher in GPSM1^-/-^ mice. These data indicate that, at least in conductive vessels, sGC function is upregulated in the absence of GPSM1. These observations are consistent with previously observed GPSM1-dependent inhibition of sGC in cellular lysates.

## Conclusion

Data presented here clearly indicate that GPSM1 is a modifier of sGC vascular function. Since GPSM1 is known to associate with Gα subunit of heterotrimeric G-proteins, it remains to be determined, if there is any GPSM1-mediated cross-talk between sGC function and heterotrimeric G-protein signaling.

